# P16 and P53 Play Distinct Roles in Different Subtypes of Breast Cancer

**DOI:** 10.1371/journal.pone.0076408

**Published:** 2013-10-11

**Authors:** Ming Shan, Xianyu Zhang, Xiaolong Liu, Yu Qin, Tong Liu, Yang Liu, Ji Wang, Zhenbin Zhong, Youxue Zhang, Jingshu Geng, Da Pang

**Affiliations:** 1 Department of Breast Surgery, The Affiliated Tumor Hospital of Harbin Medical University, Harbin, China; 2 Department of Pathology, The Affiliated Tumor Hospital of Harbin Medical University, Harbin, China; Shanghai Jiao Tong University School of Medicine, China

## Abstract

Breast cancers are heterogeneous and complex diseases, and subtypes of breast cancers may involve unique molecular mechanisms. The p16^INK4a^ and p53 pathways are two of the major pathways involved in control of the cell cycle. They also play key roles in tumorigenesis. However, whether the roles of these pathways differ in the subtypes of breast cancer is unclear. Therefore, p16 and p53 expression were investigated in different breast cancer subtypes to ascertain their contributions to these cancers. A total of 400 cases of non-invasive ductal carcinoma in situ (DCIS) and invasive ductal carcinoma (IDC), including the major molecular subtypes luminal-A, luminal-B, Her-2, and triple-negative subtypes, and 50 cases of normal controls were compared. Luminal-A cancers expressed the lowest level of p16 among the subtypes in DCIS, and the level of p16 expression was up-regulated in the luminal-A of IDC (P<0.008). Triple-negative breast cancers were characterized by a correlation of p53 overexpression with a high level of p16 expression. Luminal lesion types with high p16 expression in DCIS were found to be more likely to develop into aggressive breast cancers, possibly promoted by p53 dysfunction. Taken together, the present study suggest that p16 expression in luminal-A breast cancers is associated with their progression from DCIS to IDC, and both p53 and p16 expressions are important for the development of triple-negative breast cancers in DCIS and IDC.

## Introduction

Breast cancers are complex, heterogeneous diseases with unclear etiologies. Cell-cycle deregulation is often observed in such cancers. The cell cycle is monitored by checkpoints that ensure the integrity of the genome and the fidelity of chromosome separation via the ordered execution of cell-cycle events. Cell-cycle deregulation can lead to uncontrolled cell growth and contribute to tumor formation. Deregulated cell proliferation is characteristic of tumor cells, and gene mutations affecting control of the cell cycle are extremely common in human cancers, including breast cancer [1]. The p16INK4a (pRb/p16INK4a/cyclin D1) and p53 (p14ARF/mdm2/p53) pathways are the two main cell-cycle control pathways frequently targeted in tumorigenesis, and the alterations in each pathway depend on the type of tumor [2]. Virtually all human tumors show deregulation in both the p16 and p53 pathways, either simultaneously or consecutively [3], [4].

P16 binds to CDKs 4 and 6, inducing conformational changes that disrupt kinase interaction with D-type cyclins, thereby inhibiting CDK activation [5]–[9]. Through inactivation of CDK 4 and 6, p16 prevents phosphorylation and inactivation of Rb family cell cycle regulators. The tumor suppressor gene wild-type p53 plays a key role in many cellular pathways controlling cell proliferation, cell survival, and genomic integrity. P53 acts as a brake on proliferation when cells experience stress, such as DNA-damage, hypoxia, and oncogene activation. Disrupting p53 function, such as the inactivation that occurs in response to various p53 mutations and high-risk HPV E6 protein. This disruption promotes checkpoint defects, genomic instability, and inappropriate survival, leading to uncontrolled proliferation of damaged cells [10]. The proliferative advantage given by p53 inactivation and the ubiquitous expression of p53 explain why it is found to be mutated in almost every type of cancer [11].

Recent analyses incorporating microarray gene expression profiling offer a new method of classifying human breast cancers into subtypes, such as luminal A, luminal B, Her-2, and basal-like breast cancers [12]–[14]. Because of the cost and complexity of microarray approaches in routine practice, a surrogate immunohistochemistry (IHC) assay was used to classify tumors as luminal A (ER+ and/or PR+, HER2− or low proliferation), luminal B (ER+ and/or PR+, and either HER2+ and/or high proliferation), Her-2 (ER− and PR−, HER2+) or basal-like, which is characterized by a triple negative phenotype (ER−/PR−HER2−) [15]. The fact that these subtypes and their clinical impact on breast cancer outcomes can be determined consistently across data sets strongly suggests that they are indicative of distinct intrinsic tumor biological properties and behavior [16], [17].

Previous studies have shown that p16 and p53 are associated with breast carcinoma prognosis [18]–[27]. However, their roles in breast cancer subtypes are not well defined. Here, p16 and p53 expression were examined in different breast cancer subtypes. This may inform future investigations into the molecular mechanisms behind these cancers and improve current and potential therapeutics meant to treat these cancers.

## Materials and Methods

### Ethics Statement

This study, including the procedures for patient enrollment and recruitment, was approved by the Institutional Review Board of the Affiliated Tumor Hospital of Harbin Medical University, and all patients who participated in the study provided written informed consent.

### Patients, Case Selection, and Specimen Processing

The study population consisted of two groups of breast cancer patients. In the first group, 50 patients were randomly selected from 200 patients with invasive ductal carcinoma (IDC), which included breast cancer subtypes luminal-A, luminal-B, Her-2, and triple-negative. From a second population of 200 patients who had ductal-carcinoma in situ (DCIS), 50 patients were also selected randomly. Then, 50 otherwise healthy women whose breast tumors were benign (adenosis, intraductal papilloma, stromal fibrosis, etc.) were selected to serve as normal controls. All patients were treated at the Affiliated Tumor Hospital of Harbin Medical University, China from 2005 to 2007. All cases were processed uniformly, and samples were sectioned in a fresh state (in the normal control group, tissue distant from the benign lesion was sampled) and fixed overnight in 10% neutral buffered formalin before processing (24–33 h of formalin fixation). Formalin-fixed, paraffin-embedded tumor and control blocks were cut into 3 µm sections and stained with hematoxylin and eosin (H&E). H&E stained slides were used to review the pathology for each case. Each case diagnosis was reconfirmed by two independent pathologists (Figure S1).

### Immunohistochemistry and Fluorescence in situ Hybridization Analysis

Immunohistochemical (IHC) staining for ER and PR were performed on a Benchmark XT autostainer (Ventana Medical Systems Inc, Tucson, AZ, U.S.) using an I-View detection kit. Antibodies and other reagents were purchased as follows: ER monoclonal antibody, Ventana, AZ, U.S., catalog No. 76O–2596; PR monoclonal antibody, DAKO, Glostrup, Denmark, catalog No. M3569.

DCIS and IDC with weak, moderate, or strong nuclear labeling for ER or PR in more than 1% of cells were considered ER-positive and PR-positive, respectively (Figures S2 and S3) [28]. HER-2 IHC was performed using as DAKO Herceptest kit according to the manufacturer’s protocol. Cases were scored using the following established criteria: 0 (negative), 1+, 2+ (equivocal), and 3+ (positive) (Figure S4). Fluorescence *in situ* hybridization analysis for HER-2 amplification was performed on all cases with IHC scores of 2+ (equivocal) using a Path Vysion Kit (Des-Plaines, IL, U.S.). To qualify as HER-2 positive for this study, a case had to be classified with either a 3+ (positive) IHC score or a HER-2 fluorescence *in situ* hybridization amplification ratio greater than 2.2. The slides were also reviewed by two of the authors to confirm the interpretation.

**Figure 1:**
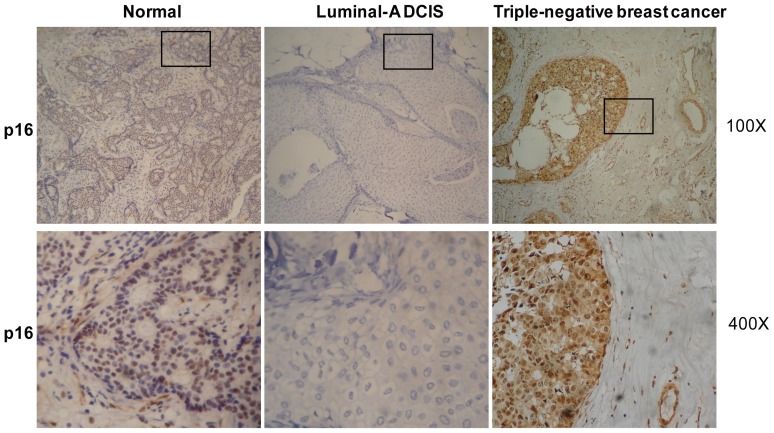
P16 was downregulated in luminal-A of DCIS but upregulated in triple-negative breast cancer. Typical images of p16 IHC staining are shown at two different magnifications: top, 100×; bottom, 400× for the boxed areas of the top images.

IHC for p16 was performed using the monoclonal antibody ab108349 (Abcam Ltd). P16 staining was scored on a scale of 0–3 based on the extent of immunopositive cells (0, no staining; 1, <25%; 2, 25–75%; 3, >75%). P16 had a predominantly cytoplasmic pattern of immunopositivity and nuclear staining, the remainder of the study focused primarily on nuclear immunostaining (Figure 1). In this manuscript, where indicated, p16 low-expression immunostaining refers to scores of “0” and “1”, and high-expression immunostaining refers to scores of = 3.

**Figure 2:**
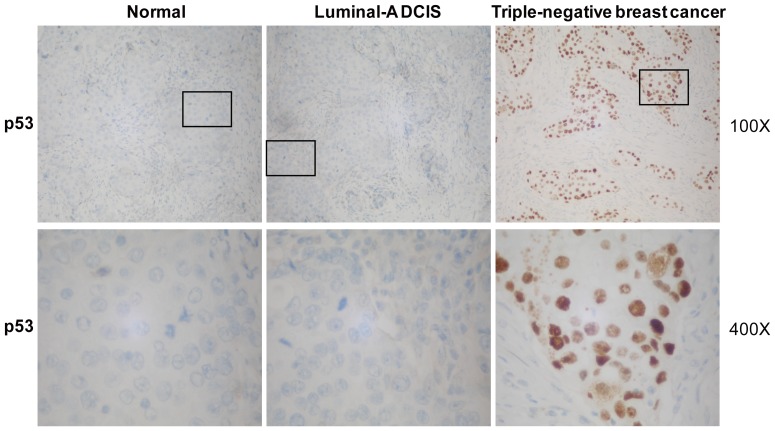
Triple-negative breast cancer samples were negative for p53 mutation. Typical images of p53 IHC staining are shown at two different magnifications: top, 100×; bottom, 400× for the boxed areas of the top images.

P53 (Ventana, monoclonal antibody, catalog No. 760–2542) and Ki-67 (Ventana, monoclonal antibody, catalog No. M7240) IHC were also performed on a Benchmark XT autostainer. For p53 and Ki-67, only nuclear labeling was scored. For p53, labeling of >30% of nuclei was considered aberrant overexpression. This was closely but not perfectly correlated with p53 mutations [29]. Labeling of <30% of nuclei was interpreted as negative for aberrant overexpression, and labeling of approximately 30% of nuclei was referred to as equivocal overexpression (Figure 2). These latter cases were excluded from statistical analyses of p53 expression. When assessing proliferation activity, it was reasoned that the mean Ki-67 index might be artificially low due to sampling of central hypoxic regions of the IDC, whereas the highest Ki-67 index of a single sample core might better reflect proliferation at the IDC’s leading edge. Therefore, for Ki-67, the mean labeling index from the periphery to the center of the sample cores was recorded. A Ki-67 cutoff point of 13% was used to designate a highly proliferating sample, and then the subtype was defined [15].

### Case Characterization

According to IHC, cases were categorized into one of 4 groups based upon accepted and previously validated IHC surrogate profiles. Each category included 50 cases of DCIS and 50 cases of IDC. Luminal A tumors were immunoreactive for ER and/or PR, negative for HER-2 or low proliferation. Tumors that were ER+ and/or PR+, and either HER2+ and/or high proliferation were considered luminal B tumors. The subtype of Her-2 was defined as ER−, PR−, and HER2+. Basal-like tumors were the most controversial type. On the basis of published criteria, all basal-like cases approximated a triple negative phenotype (ER−/PR−/HER2−). For this reason, triple-negative samples were used instead. Of note, one category that considered normal-like by gene expression profiling remained poorly defined and lacked a validated IHC surrogate profile. For this reason, group/category was not included in the remainder of the study.

### Statistical Analysis

Data were analyzed using the Student t test, the Fisher exact test, or the Mann-Whitney U test as applicable.

## Results

### P16 is Downregulated in Luminal-A of DCIS but Upregulated in Triple-negative Breast Cancer

Initially, p16 expression was investigated in different breast cancer subtypes using immunostaining (Figure 1). Eighteen luminal-A cases showed lower p16 expression than other subtypes of DCIS (*P*<0.008; Table 1). In contrast, 28 triple-negative cases in the same DCIS group showed higher p16 expression than other DCIS cases (*P*<0.008; Table 1). In the IDC group, however, p16 expression in luminal-A subtype was not significantly different from other subtypes (*P*>0.05). P16 expression in triple-negative IDC was consistent with triple-negative DCIS. Twenty-eight cases showed pronounced p16 expression, more than in other IDC subtypes (*P*<0.0001; Table 1). P16 was highly expressed in triple-negative breast cancers but downregulated in luminal-A of DCIS.

**Table 1:** P16 expression in different breast cancer subtypes.

	P16 (%)					
Pathology	0	1	2	3		*P* value
**DCIS**						
** Luminal-A**	5(10%)	13(26%)	31(62%)	1(2%)		***P*** **^a^**<0.008
** Luminal-B**	3(6%)	6(12%)	28(56%)	13(26%)		
** Her-2**	4(8%)	12(24%)	20(40%)	14(28%)		
** Triple-negative**	1(2%)	3(6%)	18(36%)	28(56%)		***P*** **^a^**<0.008
**IDC**						
** Luminal-A**	2(4%)	10(20%)	24(48%)	14(28%)		
** Luminal-B**	2(4%)	9(18%)	27(54%)	12(24%)		
** Her-2**	1(2%)	10(20%)	26(52%)	13(26%)		
** Triple-negative**	0	3(6%)	19(38%)	28(56%)		***P*** **^b^**<0.0001

**P^a^**: the corresponding subtype was compared to other types in the DCIS group.

**P^b^**: the corresponding subtype was compared to other types in the IDC group.

### P16 Downregulation Correlated with Breast Cancer Patient Characteristics

Then the issue of whether p16 expression was correlated with specific patient characteristics was assessed. In DCIS cases, low levels of p16 expression were observed in older women and women who had delayed menarche. Tumors were smaller and better differentiated by tumor grade. There were less tumor proliferation features was characteristic of patients who had low p16 expression (Table 2). However, for these characteristics, there were no differences between low and high levels of p16 expression in IDC cases (Table 2), with the exception of the patient age at diagnosis and Ki-67. Details regarding breast cancer risk factors and high or low p16 expression in DCIS and IDC are summarized in Table 2. P16 downregulation was correlated with better cancer patient outcomes, chiefly in DCIS patients.

**Table 2:** Correlation between breast cancer clinical factors and p16 expression in DCIS and IDC.

	P16 (DCIS)					P16 (IDC)		
Factors	Low expression			High expression	*P* value	Low expression	High expression	*P* value
**Patient characteristics**								
Age at diagnosis	53.04±8.85			46.82±8.78	***P = 0.00055***	52.92±10.46	49.13±8.41	***P = 0.0469***
Age at menarche	13.4±1.46			12.84±1.40	***P = 0.0476***	13.03±1.21	12.76±1.10	
BMI at diagnosis(kg/m2)	24.35±3.08			23.77±3.488		23.91±3.13	24.31±3.40	
***Frequencies***								
Family history of BC or OC	3			3		2	3	
Family history of other tumors	6			5		5	8	
History of benign mass	8			12		9	13	
**Tumor characteristics**								
**Size**					***P = 0.015***			
<2 cm	25			16		9	18	
≥2 cm	22			40		28	49	
**Grade**					***P = 0.00028***			
Well differentiated	24			11		3	2	
Moderately differentiated	11			13		34	59	
Poorly differentiated	12			32		1	6	
**Nodal involvement**								
No nodes	42			43		7	8	
1–3	5			12		23	44	
≥4	0			1		7	15	
**Ki-67**					***P = 0.00054***			***P = 0.0409***
<13%		36	24			17	26	
≥13%		11	32			20	41	

**BMI**, body mass index; **BC**, breast cancer; **OC**, ovary cancer.

### P16 Might Contribute to Subsequent Development of Advanced Breast Cancer in DCIS

To determine whether p16 tumor suppression has a role in DCIS lesions with respect to subsequent tumor development, p16 expression was measured in 41 cases of luminal type DCIS and subsequent development of advanced cancer was monitored for more than 6 months. All patients in this category received no treatment except endocrine therapy after surgery. A subsequent tumor event (recurrence) was defined as a subsequent DCIS lesion or invasive cancer lesion as diagnosed in the ipsilateral breast or at a distant site at least 6 months after the initial DCIS diagnosis.

**Figure 3:**
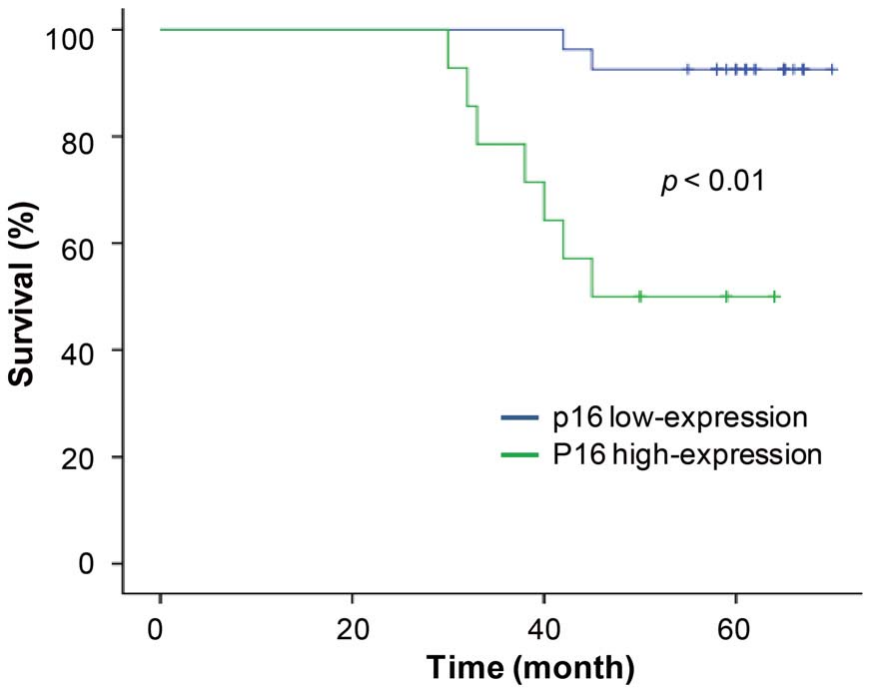
P16 expression predicted the risk of subsequent advanced cancer development among women with luminal DCIS. *P* = 0.001, low-p16-expression DCIS versus high-p16-expression DCIS (log-rank test).

In the 41 DCIS cases, approximately one-third of DCIS lesions were highly stained for p16 staining and the remaining cases showed low staining intensity for p16. Patients with high levels of p16 expression were found to be more likely to develop subsequent advanced breast cancer than patients with low p16 expression (Figure 3). These data indicate that p16 may contribute to subsequent cancer development in DCIS.

### P16 and P53 Play Distinct Roles in Different Subtypes of Breast Cancer

To determine the potential mechanism behind DCIS with high p16 expression and the propensity of these cases to develop advanced breast cancer, the p53 status of these cases was determined. Correlations with p16 expression and p53 aberrant overexpression (Figure 2). In low p16 expression groups in DCIS cases, most of the luminal-A subtypes were negative for p53 aberrant overexpression, as were one-third of luminal-B subtypes and half of the Her-2 subtypes. No DCIS triple-negative subtypes were negative for p53 overexpression. Of the IDC cases, most of the luminal-A subtypes were negative for p53, as were almost half of luminal-B subtypes, the majority of Her-2 subtypes, and almost all triple-negative IDC subtypes (Table 3). In this way, in DCIS, but not IDC, low p16 expression was accompanied by an absence of p53 aberrant overexpression in the luminal-A subtype (*P*<0.007). Table 3 depicts the remainder of these findings. In this way, aberrant overexpression of p53 was found to be related to high p16 expression in DCIS and IDC triple-negative subtypes (*P*<0.007). Because as p53 mutation is closely correlated with aberrant overexpression of p53 [29]–[31], these results might indicate that the dysfunction of the p53 pathway chiefly occurs in triple-negative breast cancers.

**Table 3:** P16 pathways of different groups correlated to the p53 pathway.

	P53		
Low P16 expression	−	+	*P* value
**DCIS**			
Luminal-A	17	1	***P*** **<0.007**
Luminal-B	3	6	
Her-2	8	8	
Triple-negative	0	4	
**IDC**			
Luminal-A	11	1	
Luminal-B	4	7	
Her-2	8	3	
Triple-negative	2	1	
**High P16 expression**			
**DCIS**			
Luminal-A	1	0	
Luminal-B	10	3	
Her-2	10	4	
Triple-negative	6	22	***P*** **<0.007**
**IDC**			
Luminal-A	12	2	
Luminal-B	9	3	
Her-2	10	3	
Triple-negative	7	21	***P*** **<0.007**

## Discussion

In the present study, in DCIS, p16 was found to be more downregulated (low-expression) in luminal-A cancer subtypes than in any other breast cancer subtype studied here. P16 was upregulated (high-expression) in triple-negative subtypes, which is consistent with previous reports [32], [21]. In IDC, triple-negative subtypes showed more positive p16 immunostaining than other subtypes. However, low expression of p16 was not observed in luminal-A, unlike in DCIS. The reason for this difference was unclear [21], [33]–[35]. P16 may be a canonical regulator of cell proliferation and apoptosis. For this reason, its expression changed along with changes to the cell cycle. To determine p16 dysfunction, p16 IHC was classified by cancer subtype, and these samples were compared to normal mammary tissue (Tables S1 and S2). In DCIS, luminal-A showed less p16 expression than other subtypes and p16 IHC was more strongly stained in triple-negative subtypes than in other subtypes, both in true DCIS and IDC cases. No anomalous p16 expression phenotypes were observed among IDC luminal-A subtypes, unlike DCIS cases. This may mean that an early (induction) phase of carcinogenesis was observed here and that luminal-A breast cancer is induced by the abrogated p16 pathway [36], [37]. During the invasion stage, luminal-A breast cancer may have more complicated regulation signaling, such as negative feedback regulation pathways [25]. In this way, the initial factor within the abnormal p16 pathway may have been masked by other dysfunctions.

Correlation analysis between p16 and p53 in different breast cancer subtypes revealed that, when p16 was downregulated, only DCIS luminal-A subtypes were frequently negative for IHC p53 staining. Even with upregulated p16, triple-negative subtypes of both DCIS and IDC showed considerable p53-positive staining, which was consistent with previous reports [38], [39]. These data suggest that p16 dysfunction could initiate luminal-A subtypes. In contrast, p53 might play an important role in triple-negative breast cancer development [40], [41].

A comparison of breast cancer risk factors, breast tumor characteristics, and p16 expression in DCIS and IDC cases revealed that low levels of p16 expression in DCIS cases was closely correlated with advanced age, later menarche, smaller tumor size, well-differentiated tumors, and low cell proliferation rates. High levels of p16 expression were correlated with opposing factors in DCIS cases [21], [19]. However, these observations were not made for IDC cases. In IDC cases, low levels of p16 expression were correlated with advanced age and Ki-67-positive cells. It was hypothesized that p16 expression might change during tumor development and that p16 acts as an initiator. To confirm this, the prognoses of patients with low p16 expression were compared to those of patients with high p16 expression in luminal subtypes of DCIS. Patients with low levels of p16 expression had better outcomes and prognoses, so p16 may be a measurable risk factor for these cancer subtypes. Gauthier and co-workers suggested that p16 could be one of several useful prognostic markers [25]. It may be used to detect early cancers and prevent overtreatment of certain subtypes.

In conclusion, p16 and p53 play distinct roles in different breast cancer subtypes. P16 is visibly important in luminal cancer subtypes, especially the luminal-A type. This marker is closely correlated with good prognosis. However, p53 plays an important role in triple-negative subtypes, suggesting unique molecular mechanisms within each cancer subtypes. These data should inform future studies into these cancers and improve cancer therapeutic strategies for all breast cancer patients.

## Supporting Information

Figure S1 **H&E Staining.** Two different magnifications: top, 100×; bottom, 400× for the boxed areas of the top images.
(TIF)

Figure S2 **ER IHC Staining.** Two different magnifications: top, 100×; bottom, 400× for the boxed areas of the top images.
(TIF)

Figure S3 **PR IHC Staining.** Two different magnifications: top, 100×; bottom, 400× for the boxed areas of the top images.
(TIF)

Figure S4 **HER-2 IHC Staining at 4 Grades.** Two different magnifications: top, 100×; bottom, 400× for the boxed areas of the top images.
(TIF)

Table S1 Low expression and high expression of p16 in luminal-A breast cancers and normal tissues.
(DOC)

Table S2 Low expression and high expression of p16 in triple-negative breast cancers and normal tissues.
(DOC)
