# Supplementary figures and images for: P16 and P53 Play Distinct Roles in Different Subtypes of Breast Cancer
Source: PLoS One. 2013 Oct 11;8(10):e76408. doi: 10.1371/journal.pone.0076408 (PMC3795768; doi:10.1371/journal.pone.0076408)

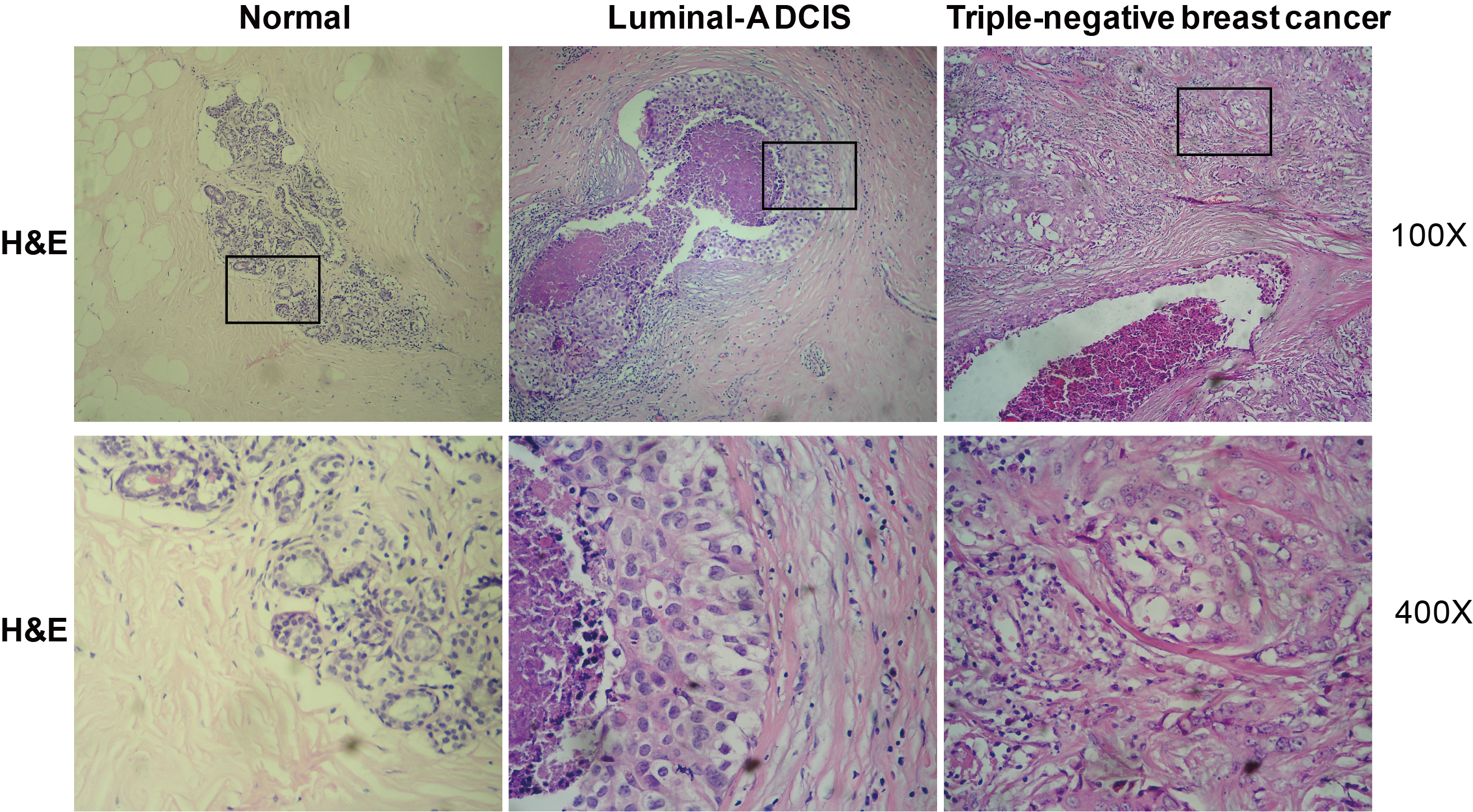

Supplement: Figure S1 — H&E Staining. Two different magnifications: top, 100×; bottom, 400× for the boxed areas of the top images. (TIF) [file pone.0076408.s001.tif]

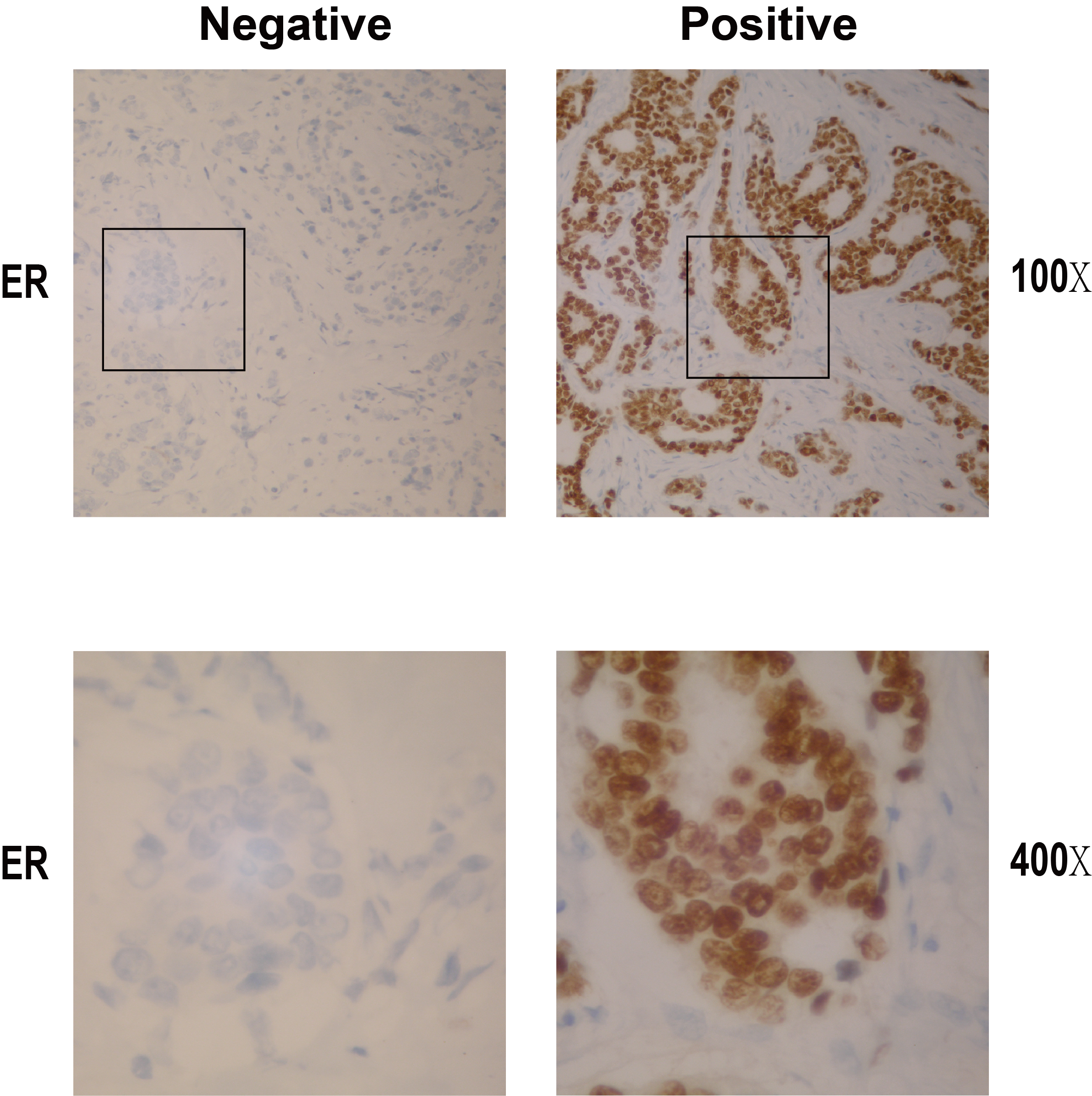

Supplement: Figure S2 — ER IHC Staining. Two different magnifications: top, 100×; bottom, 400× for the boxed areas of the top images. (TIF) [file pone.0076408.s002.tif]

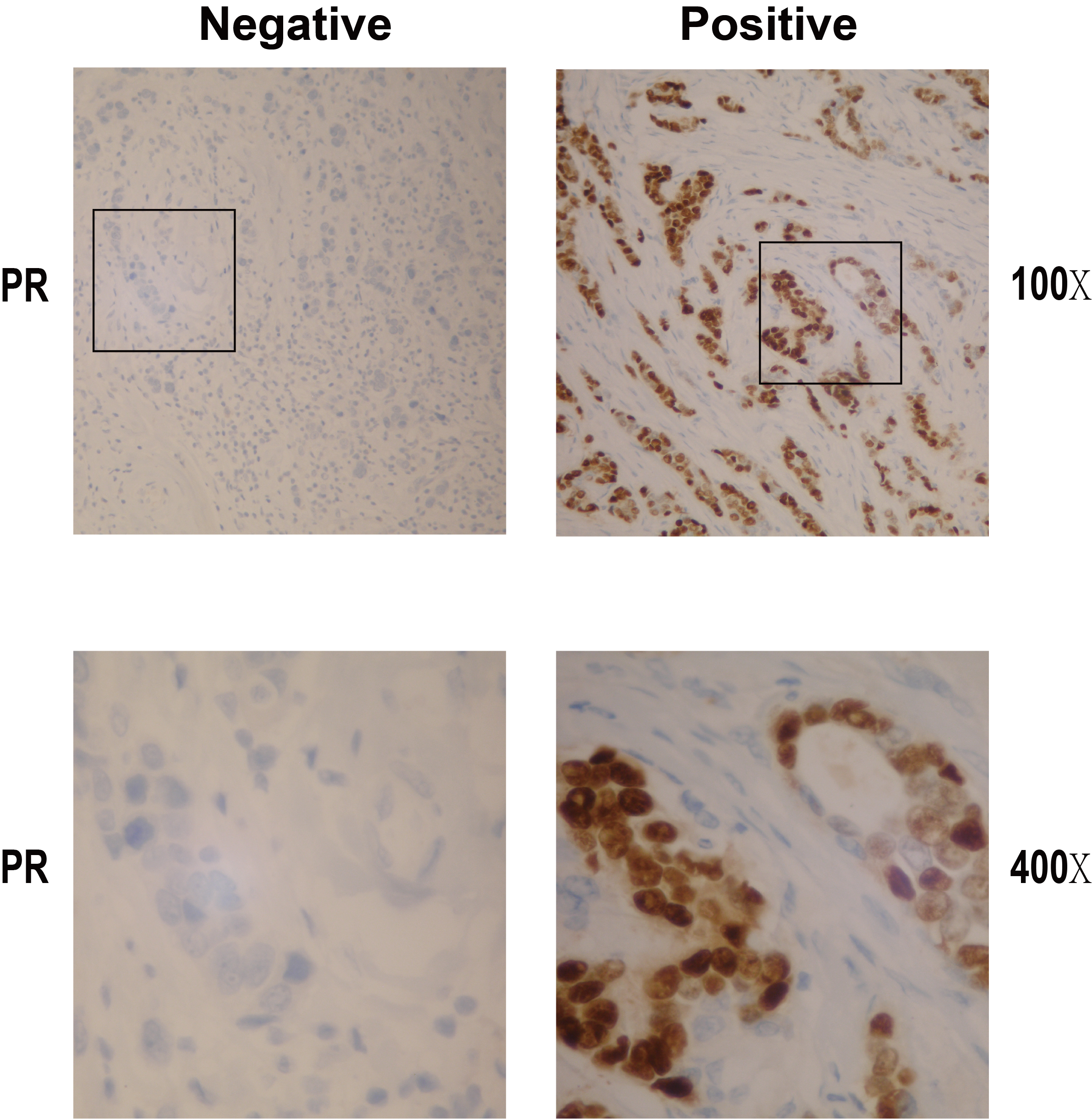

Supplement: Figure S3 — PR IHC Staining. Two different magnifications: top, 100×; bottom, 400× for the boxed areas of the top images. (TIF) [file pone.0076408.s003.tif]

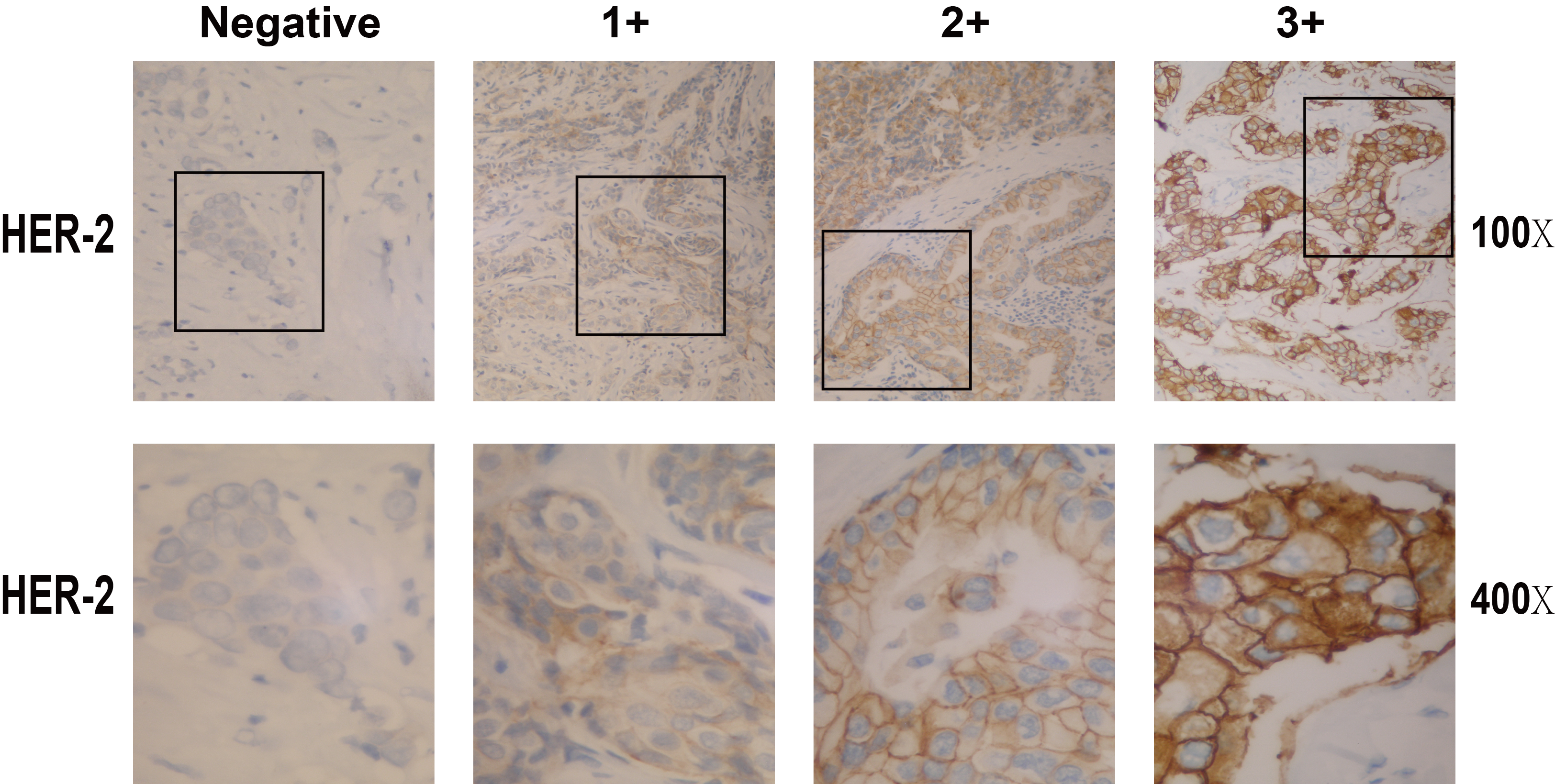

Supplement: Figure S4 — HER-2 IHC Staining at 4 Grades. Two different magnifications: top, 100×; bottom, 400× for the boxed areas of the top images. (TIF) [file pone.0076408.s004.tif]
