# Supplementary material for: P16 and P53 Play Distinct Roles in Different Subtypes of Breast Cancer
Source: PLoS One. 2013 Oct 11;8(10):e76408. doi: 10.1371/journal.pone.0076408 (PMC3795768; doi:10.1371/journal.pone.0076408)
Supplement: Table S1 — Low expression and high expression of p16 in luminal-A breast cancers and normal tissues. (DOC) [file pone.0076408.s005.doc]

**Table S1**

“Low-expression” and “high-expression” of p16 in luminal-A breast cancers and normal tissues

|  | **P16** | |  | |
| --- | --- | --- | --- | --- |
| **Pathology** | **Low-expression** | **High-expression** | **P value** | |
| **DCIS-Luminal-A** | 18 | 1 | **P^a^﹤0.0125** | |
| **IDC-Luminal-A** | 12 | 14 | **P^b^﹤0.01** | |
| **Normal** | 7 | 6 |  |  |

**P^a^**: the luminal-A in DCIS and in IDC compared with normal control.

**P^b^**: the comparison between the luminal-A in DCIS and in IDC.
